# Correspondence

**Published:** 1869-10

**Authors:** P. G. Kelsey

**Affiliations:** Fort Wayne, Ind.


### Correspondence.

Fort Wayne, Ind., *August 25th, 1869.*

Dr. N. S. Davis, *Chicago, Ill.:*

Dear Doctor:—Having recently made use of carbolic acid for the destruction of maggots, inhabiting a locality which rendered their mechanical removal impracticable, with good results, and never having seen an account of the same application, I am moved to give an account of my experiment.

On the 4th inst., I was called to a case of *epithelial carcinoma*, in which the soft parts of the nose had been entirely destroyed by the insidious disease, which had also penetrated far into the nasal fossa, and rendered the poor sufferer an object of pitiful disgust by its terrible work; and now, as if this was not enough, she had fallen victim to the *fly*, and was verily *food for the worms while she yet lived.* The sight was indeed most sickening; for the left nasal fossa, laid open and gaping from the removal of the soft parts, *was completely filled with maggots*, of large size, some of them being half an inch in length, and all, with their accustomed activity reveling on human flesh. To add to the disgust, one would occasionally come wriggling out of the patient's mouth; and the left eye, the sight of which was gone, was also filled with these loathsome things; so that it would seem that the entire face was alive with them. Whether the nasal duct had been enlarged for their convenience, or whether they were of a separate deposit in the eye, I know not.

The patient was a Frenchwoman, about 50 years of age, and had been afflicted for a number of years; but this once being the only time I saw her, I can give no history of the case, and none is needed. At this time, she was greatly prostrated, and when undisturbed by attendants, she did not suffer much pain, and manifested but little consciousness, whereas, a day or two before the maggots were noticed, her suffering was intense. Now, the removal of these intruders by the usual means was rendered out of the question, by the extreme sensativeness of every part of the patient's face, which forbade a touch even; and to make no effort for their removal, even though I knew her to be dying, would seem criminal to her friends. Consequently, I ordered an anodyne, to aid her in bearing the attempt, and for the destruction of the maggots; as a vermifuge, I ordered a solution of carbolic acid, 20 gr. to the ℥; to be applied greatly reduced at first, the strength to be increased as it could be borne. The result of this was eminently satisfactory; two or three applications not only destroying and removing every maggot, but also otherwise cleansing and purifying the foul sore in a remarkable degree, which was followed by general improvement, so that the patient rallied, partook again of nourishment, and for three or four days seemed a great deal better.

A word with regard to the use of carbolic acid, as a wash for indolent ulcers. A case of two years standing, very severe, at times threatening the patient's life, which had bade defiance to almost every thing—carbolic acid included, used very strong—is now yielding and healing kindly under a very weak application of the acid. Of a solution of 20 grs. to the ℥, only 15 or 20 drops are added to a teacupful of water, and this is applied twice a day.

P. G. KELSEY., M.D.

